# How to establishing an indicators framework for evaluating the performances in primary TB control institutions under the new TB control model? Based on a Delphi study conducted in Guangxi, China

**DOI:** 10.1186/s12889-022-14865-4

**Published:** 2022-12-27

**Authors:** Tengyan Wu, Huimin He, Suosu Wei, Pinghua Zhu, Qiming Feng, Zhong Tang

**Affiliations:** 1 Department of Health Service Management, School of Information and Management, Guangxi Medical University, Nanning, China; 2 Editorial Board of Chinese Journal of New Clinical Medicine, The People’s Hospital of Guangxi Zhuang Autonomous Region, Nanning, China

## Abstract

**Background:**

In China, the new TB control model of trinity form had been implemented in all parts, and the comprehensively evaluation to the performances in primary TB control institutions were closely related to the working capacity and quality of TB service, but there was still no an unified evaluation indicators framework in practice and few relevant studies. The purpose of this study was to establish an indicators framework for comprehensively evaluating the performances in primary TB control institutions under the new TB control model of trinity form in Guangxi, China.

**Methods:**

The Delphi method was used to establish an indicators framework for comprehensively evaluating the performances in primary TB control institutions under the new TB control model of trinity form, and the analytic hierarchy process(AHP) was used to determine the weights of all levels of indicators, from September 2021 to December 2021 in Guangxi, China.

**Results:**

A total of 14 experts who had at least 10 years working experience and engaged in TB prevention and control and public health management from health committee, CDC, TB designated hospitals and university of Guangxi were consulted in two rounds. The average age of the experts were (43.3 ± 7.549) years old, and the effective recovery rate of the questionnaire was 100.0%. The average value of authority coefficient of experts (Cr) in the two rounds of consultation was above 0.800. The Kendall’s harmony coefficient (W) of experts’ opinions on the first-level indicators, the second-level indicators and the third-level indicators were 0.786, 0.201 and 0.169, respectively, which were statistically significant (*P* < 0.05). Finally, an indicators framework was established, which included 2 first-level indicators, 10 second-level indicators and 37 third-level indicators. The results of analytic hierarchy process (AHP) showed that the consistency test of all levels of indicators were CI < 0.10, which indicating that the weight of each indicator was acceptable.

**Conclusion:**

The indicators framework established in this study was in line with the reality, had reasonable weights, and could provide a scientific evaluation tool for comprehensively evaluating the performances in primary TB control institutions under the new TB control model of trinity form in Guangxi, China.

**Supplementary Information:**

The online version contains supplementary material available at 10.1186/s12889-022-14865-4.

## Introduction

**Fig. 1:**
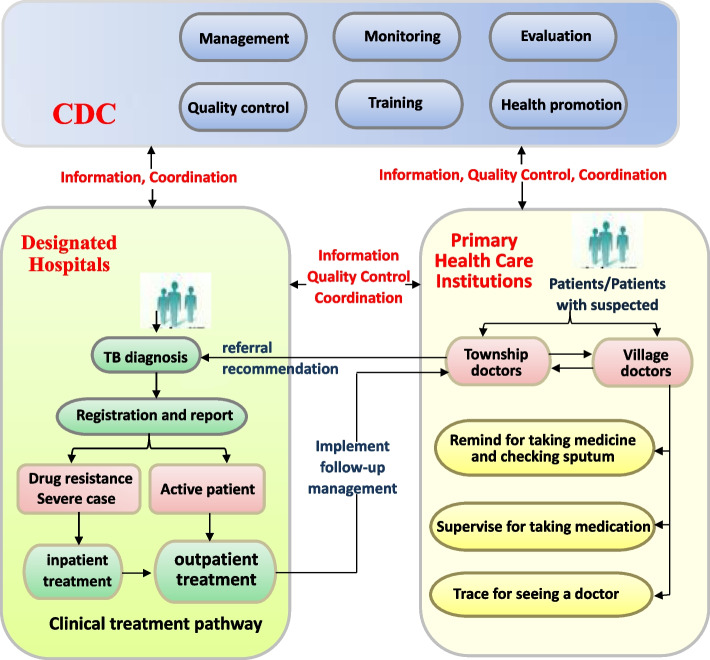
The new TB control model of trinity form in Guangxi, China

Tuberculosis (TB) was a major public health problem in the world [1, 2]. In order to deal with the challenge of TB, governments of all countries had established TB control model in line with their national conditions, and actively carried out actions around the work objectives of each stage to achieve the goal of “End TB”. China was one of the 30 countries with high TB burden in the world, and the situation of TB control was severe. Implementing anti-TB institutions cooperation was the basic strategy for TB control in China. Since 2010, the new TB control model of trinity form had been implemented gradually in all regions [3]. In the new TB control model, the Centers for Disease Control and Prevention (CDC) was responsible for planning and management, the designated hospitals was responsible for diagnosis and treatment of patients, and the primary health care institutions was responsible for tracking and management of patients, that combine into the trinity form of “the anti-TB institutions cooperation which responsible for TB prevention, treatment and management together”. See the Fig. 1. The new TB control model of trinity form was expanded from the previously model that TB control being undertook by CDC alone. Under the new TB control model, the primary TB control institutions (including CDC in county-level and the designated hospitals in county-level and the primary health care institutions) undertook specific work that discovery, report, diagnosis, treatment and management of TB patients, and played an important role. The work in the primary TB control institutions involved multiple departments and interests, and was an organic whole. So the comprehensive evaluation should be carried out based on an indicators framework, in order to improve the working capacity of the primary TB control institutions. Especially after the COVID-19 pandemic [4–6], it was of great practical significance to carry out comprehensive evaluation of the performances in primary TB control institutions in the process of restoring the service supply of TB control and accelerating to realization the global goal of “End TB”. However, there was no an unified indicators framework for carrying out the comprehensively evaluation to the performances in primary TB control institutions, and there were few relevant studies in China.

Therefore, in this study, Delphi method and analytic Hierarchy Process (AHP) were used to establish an indicators framework for comprehensively evaluating the performances in primary TB control institutions under the new TB control model of trinity form, in order to provide theoretical and evidence-based basis for carrying out the comprehensive and effective evaluation of TB control in various in Guangxi and the other regions of China.

## Materials and methods

### Study site

The Guangxi Zhuang Autonomous Region (Guangxi), with a population of 50 million, was an underdeveloped southwestern region where the TB epidemic was relatively serious in China [7–9]. At present, the new TB control model of trinity form had been implemented in all areas of the region in Guangxi. Previous studies had shown that there were many problems and difficulties in TB control of Guangxi, such as insufficient financial investment, weak ability in primary TB control institutions, common delay in treatment of patients, heavy disease economic burden and so on. After the impact of the epidemic of COVID-19, TB control would face more severe challenges. There was an urgent need to comprehensively evaluate the performance in primary TB control institutions based on a scientific indicators framework, so as to provide reference for the continuous improvement the quality of TB control. Our study was conducted in Guangxi with the period from September 2021 to December 2021.

### Selection of experts

Using objective sampling method, 14 experts who had at least 10 years working experience and engaged in TB prevention and control and public health management were selected from health committee, CDC, TB designated hospitals and university of Guangxi, China.

### Establishing of the initial indicators framework

Taking “tuberculosis (TB)”, “indicators framework”, “evaluation” as the key word, the relevant literature were searched online in PubMed, Embase, Cochrane Lirary databases, China national knowledge infrastructure(CNKI), Wanfang Database and so on. The relevant policy and regulation and reports from the year 2011 to 2021 were consulted on the official websites of national and local health commissions and CDC. And then, an initial indicators framework including 2 first-level indicators,10 second-level indicators and 69 third-level indicators were established after discussing by the research group.

### Screening indicators by Delphi method

Delphi method, also known as expert survey method, was initiated and implemented by the RAND Corporation in 1946 [10, 11]. Later, the method was widely used in business, military, education, health care and other fields, and showed its superiority and applicability in the process of use [12]. The Delphi method was essentially a feedback anonymous letter inquiry method. Its general process was: after obtaining the opinions of experts on the problem to be predicted, the result would be sorted out, summarized and made statistics, and then gave anonymous feedback to the experts, asked for opinions again, concentrated and gave feedback again, until a consensus was got. Delphi method had three characteristics, that were anonymity, multiple feedback, group statistical response [10–13]. The characteristics of Delphi method made it the most effective judgment and prediction method, and the results were widely representative and reliable.

Two rounds of questionnaires consultation through email were carried out in the study. In the first round of consultation, the contents of the questionnaire included: the purpose of consultation, the basic information of the experts, the explanation of the indicators, and the opinion column for the modification or deletion were setting to collect the opinions from the experts. The second round of consultation was designed basing on the statistical summary of the first round of consultation results. After two rounds of consultation, the consensus was got in this study.

### Quantitative assignment of options

The degree of importance, degree of familiarity and judgment basis of indicators were quantitative assignment separately by the following standards. ① Degree of importance: “Unimportant” was set as 1, “not too important” as 2, “moderately important” as 3, “Relatively important” as 4, and “Very important” as 5. ② Degree of familiarity: “unfamiliar” was set as 0.2, “not very familiar” as 0.6, “relatively familiar” as 0.8, and “very familiar” as 1.③ Judgment basis: it was divided into “theoretical analysis”, “practical experience”, “reference to domestic and foreign materials” and “intuition” according to the conventional; and the degree of influence for those above were divided into large, medium and small, respectively, and being given different quantitative values. See the Table 1.

**Table 1:** Quantitative assignment of the judgment basis and the degree of influence

The judgment basis	The degree of influence		
	Large	Medium	Small
Theoretical analysis	0.3	0.2	0.1
Practical experience	0.5	0.4	0.3
Reference to domestic and foreign Materials	0.1	0.1	0.05
Intuition	0.1	0.1	0.05
Total	1	0.8	0.5

### Evaluation parameters of the results

There were four parameters. ①Coefficient of active participation of experts: it was expressed by the recovery rate of questionnaire to evaluate the experts’ concern for the study. ②Authority coefficient of experts(Cr) : it was calculated according to the score of expert’s judgment (Ca) and the score of expert’s familiarity (Cs) to evaluate the value of experts’ advice, the calculation formula was Cr=(Ca + Cs)/2. ③Degree of concentration of experts’ opinions: It was expressed by the mean of quantitative assignment of the degree of importance and the full score ratio to evaluate the importance of indicators. ④Degree of coordination of experts’ opinions: It was expressed by the coefficient of variation (CV) and Kendall’s harmony coefficient (W) to evaluate the consistency of experts’ opinions.

### Selection criteria of indicators

Three criteria were formulated for selecting indicators.①The mean of importance was above 4.30; ②The authority coefficient was above 0.800; ③The coefficient of variation(CV) was less than 0.200; Besides, it was combined with the expert’s opinion and the working practice at the same time, then to determine the choice of indicators.

### Data analysis

The software Microsoft Excel was used to input data of consultation, and the software IBM SPSS Statistics for Windows, Version 25.0 was used to conduct statistical analysis on the results, the methods including descriptive statistic and Kendall’s W test, etc. A *p*-value < 0.05 was considered significant. The software Yaahp10.3 was used for Analytic Hierarchy Process (AHP), the processes including drawing the hierarchical structure model diagram, determining the scale of Saaty according to the mean of importance of indicators in the second round results, establishing a judgment matrix, using group decision-making, conducting consistency test, and finally determining the combination weights of indicators at all levels.

## Results

### Basic information of experts

A total of 14 experts were included in this study, with an average age of (43.3 ± 7.549) years old, the distribution of gender, organization, professional title, and degree of education of experts were showed in the Table 2.

**Table 2:** Basic information of experts (*n* = 14)

Category	n	%	Category	n	%
Gender			Professional title		
Man	10	71.4	Senior	4	28.6
Female	4	28.6	Deputy senior	7	50.0
Organization			Intermediate	3	21.4
Health Committee	2	14.3	Degree of education		
Medical Institutions	4	28.5	Doctoral Degree	4	28.6
CDC	6	42.9	Master Degree	3	21.4
University	2	14.3	Bachelor Degree	7	50.0

### Coefficient of active participation of experts

In each round of consultation, 14 questionnaires were distributed and 14 valid questionnaires were recovered, with the effective rate of 100.0% in both rounds.

### Authority coefficient of experts (cr)

The results of the first round of consultation showed that the values of Cr of the 2 first-level indicators were 0.921 and 0.904, respectively; the values of Cr among 10 s-level indicators range from 0.803 to 0.914, with an average value of (0.877 ± 0.034); the values of Cr among 69 indicators of third-level range from 0.796 to 0.907, with an average of(0.848 ± 0.285); The results of the second round of consultation showed that the values of Cr among 39 indicators of third-level range from 0.802 to 0.900, with an average value of (0.860 ± 0.244).

### Degree of concentration of experts’ opinions

The results of the first round of consultation showed that the mean of importance of the 2 first-level indicators were 5.00 and 4.21, the standard deviation were 0.000 and 0.426, and the full score ratio were 100.0% and 21.4%, respectively; The mean of importance among 10 s-level indicators range from 4.29 to 5.00, the standard deviation was 0.000 to 0.726, and the full score ratio was 42.9–100.0%; The mean of importance among 69 indicators of third-level range from 3.86 to 4.93, the standard deviation was 0.267 to 1.167, and the full score ratio was 21.4–92.9%, and the mean of importance among 23 indicators were less than 4.30. The results of the second round of consultation showed that the mean of importance among 39 indicators of third-level range from 3.93 to 5.00, the standard deviation was 0.000 to 1.072, and the full score ratio was 35.7–100.0%, and the mean of importance among 3 indicators were less than 4.30.

### Degree of coordination of experts’ opinions

The results of the first round of consultation showed that the coefficient variation (CV) of the 2 first-level indicators were 0.000 and 0.101, respectively, and Kendall’s harmony coefficient (W) was 0.786 (x^2^ = 11.000, *P* = 0.001); The variable coefficient (CV) among 10 s-level indicators range from 0.000 to 0.169, Kendall’s harmony coefficient (W) was 0.201 (x^2^ = 25.323, *P* = 0.003); The variable coefficient (CV) among 69 indicators of third-level range from 0.054 to 0.303, Kendall’s harmony coefficient (W) was 0.161 (x^2^ = 152.869, *P* = 0.000), and the value of CV among 30 indicators were above 0.200. The results of the second round of consultation show that the variable coefficient (CV) among 39 indicators of third-level range from 0.000 to 0.273, Kendall’s harmony coefficient (W) is 0.169 (x^2^ = 89.928, *P* = 0.000), and the value of CV among 2 indicators were above 0.200.

### Results of selection of indicators

According to the selection criterion of indicators which had been formulated, after the consultation of the first round, there are 30 third-level indicators were deleted; After the consultation of the second round, there were 2 third-level indicators are deleted; At last, an indicators framework was established, which included 2 first-level indicators, 10 second-level indicators and 37 third-level indicators. See the Table 3.

### Result of weights of indicators

The results of analytic hierarchy process(AHP) showed that the consistency test of all levels of indicators are CI < 0.10, which indicating that the weight of each indicator was acceptable. The weights of the first-level indicators were the combined weights. But the weights of second-level and third-level indicators were the relative weights. The calculation formula for the combined weight of the second-level and third-level indicators were: the combined weight of the second-level indicator = the combined weight of the first-level indicator *the relative weight of second-level indicator; The combined weight of the third-level indicator = the combined weight of the second-level indicator *the relative weights of third-level indicator. See the Table 3.

**Table 3:** The importance and the weights of indicators at all levels in the framework for comprehensively evaluating the performances in primary TB control institutions

Indicators	Importance		Weights	
	Scores($\stackrel{-}{x}\pm s$)	CV	RW	CW
**1 Objective performances** ^a^	5.00 ± 0.000	0.000	0.5444	0.5444
** 1.1 Discovery of patients**	4.79 ± 0.579	0.121	0.1364	0.0743
1.1.1 Examination rate of sputum smear	4.93 ± 0.270	0.054	0.1502	0.0112
1.1.2 Detection rate of positive patients	4.57 ± 0.760	0.165	0.1388	0.0103
1.1.3 Screening rate of drug resistance	4.79 ± 0.430	0.089	0.1456	0.0108
1.1.4 Screening rate of close contacts	4.64 ± 0.500	0.107	0.1418	0.0105
1.1.5 Referral rate of presumptive TB over the hospital	4.79 ± 0.430	0.089	0.1454	0.0108
1.1.6 Referral rate of presumptive TB within the region	4.64 ± 0.500	0.107	0.1410	0.0105
1.1.7 Overall attendance rate of presumptive TB	4.50 ± 0.650	0.145	0.1372	0.0102
** 1.2 Report of patients**	4.86 ± 0.363	0.075	0.1169	0.0636
1.2.1Timely reporting rate	4.57 ± 0.650	0.141	0.5046	0.0321
1.2.2 Missing reporting rate	4.50 ± 0.650	0.145	0.4954	0.0315
** 1.3 Registration of patients**	4.71 ± 0.611	0.130	0.1245	0.0678
1.3.1Registration rate of referral	4.21 ± 0.800	0.190	0.2443	0.0166
1.3.2 Registration rate of therapy	4.50 ± 0.760	0.169	0.2571	0.0174
1.3.3 Registration rate of outpatients	4.36 ± 0.740	0.171	0.2485	0.0168
1.3.4 Registration rate of screening	4.36 ± 0.740	0.171	0.2501	0.0170
** 1.4 Treatment of patients**	5.00 ± 0.000	0.000	0.1345	0.0732
1.4.1 Standard diagnosis rate of negative patients	4.93 ± 0.270	0.054	0.1725	0.0126
1.4.2 Diagnosis expert team for negative patients	4.36 ± 0.630	0.145	0.1518	0.0111
1.4.3 Utilization rate of the standard treatment scheme	4.64 ± 0.630	0.136	0.1622	0.0119
1.4.4 Receiving rate of treatment	5.00 ± 0.000	0.000	0.1755	0.0129
1.4.5 Cure rate of positive patients	4.79 ± 0.430	0.089	0.1676	0.0123
1.4.6 Successful treatment rate of patients	4.86 ± 0.360	0.075	0.1705	0.0125
** 1.5 Management of patients**	4.71 ± 0.611	0.130	0.1247	0.0679
1.5.1 Rate of regular medication	5.00 ± 0.000	0.000	0.3528	0.0240
1.5.2 Rate of scheduled return visits	4.64 ± 0.500	0.107	0.3236	0.0220
1.5.3 Attendance rate of tracking	4.64 ± 0.500	0.107	0.3236	0.0220
** 1.6 Health Education**	4.50 ± 0.650	0.145	0.1186	0.0646
1.6.1Completion rate of health education	4.43 ± 0.650	0.146	1.0000	0.0646
** 1.7 Quality Control**	4.71 ± 0.469	0.099	0.1238	0.0674
1.7.1 Establish of laboratory internal quality control	4.79 ± 0.430	0.089	0.1163	0.0078
1.7.2 Set up full-time laboratory personnel	4.43 ± 0.760	0.171	0.1070	0.0072
1.7.3 Qualified rate of sputum smear	5.00 ± 0.000	0.000	0.1218	0.0082
1.7.4 Laboratory complies with biosafety	4.79 ± 0.430	0.089	0.1161	0.0078
1.7.5 Outpatient procedures is formulate	4.64 ± 0.500	0.107	0.1125	0.0076
1.7.6 Inpatient ward is divided	4.50 ± 0.520	0.115	0.1086	0.0073
1.7.7 Funds for infection control is allocated	4.29 ± 0.730	0.169	0.1030	0.0069
1.7.8 Drug storage is up to standard	4.50 ± 0.650	0.145	0.1084	0.0073
1.7.9 Adverse drug reactions reporting is established	4.36 ± 0.740	0.171	0.1062	0.0072
** 1.8 Training and Coordination**	4.50 ± 0.650	0.145	0.1206	0.0657
1.8.1 Completion rate of training tasks	4.43 ± 0.760	0.171	0.3436	0.0226
1.8.2 Coordination mechanism is established	4.36 ± 0.840	0.193	0.3328	0.0218
1.8.3 Annual training rate for physicians	4.21 ± 0.800	0.190	0.3236	0.0212
**2 Subjective effect** ^b^	4.21 ± 0.426	0.101	0.4556	0.4556
** 2.1 Satisfaction rate of medical staff**	4.36 ± 0.633	0.145	0.4986	0.2272
2.1.1 Satisfaction rate about the treatment of work	4.50 ± 0.940	0.209	1.0000	0.2272
** 2.2 Satisfaction rate of patients**	4.29 ± 0.726	0.169	0.5014	0.2284
2.2.1 Satisfaction rate about the costs of treatment	4.36 ± 0.630	0.145	1.0000	0.2284

CV was “coefficient of variation”. RW was “relative weights”. CW was “combined weights”

^a^ Data sources of objective performances indicators include: National TB Management Information System, TB laboratory record, daily work record for TB control, etc

^b^ Data sources of subjective effect include: investigation results of medical staff and patients

## Discussion

Delphi method was a mature and easy subjective decision-making technology after years of development. It was widely used in the field of medical and health services. In the process of establishing the indicators framework, this method could synthesize the theory and practical experience of experts from different professional backgrounds to make comprehensive judgement and evaluation [10–13]. This study was based on the existing research theories and methods [14–18]. Firstly, the initial indicators framework was established based on literature review, which had a good theoretical basis. Then, 14 experts were invited to take part in Delphi expert consultation, experts come from different organizations such as health committee, CDC, TB designated hospitals and universities in Guangxi, who engaged in TB control and public health management, with bachelor’s degree or above, and 78.6% of who had deputy senior professional titles or above, which showed that the experts had good representation and abundant professional knowledge, practical skills or management experience. In the two rounds of consultation, the coefficient of active participation of experts was 100.0%, which indicating that the enthusiasm of experts to participate in this study was high. The authority coefficient of experts (Cr) showed the experts’ theoretical understanding and practical experience about the indicators, and it was generally considered that when the value of Cr was equal to or above 0.7 was accepted. In this study, the average value of Cr in the two rounds of consultation was above 0.8, which showed that the opinion of experts had a good guidance for establishing the indicators framework. After each round of consultation, indicators were screened according to the selection criteria of indicators which had been formulated before, and the opinions of experts were summarized and feedback. Finally, after two rounds of consultation, the experts’ opinions tended to be consistent and meet the requirements of Delphi method, and the indicators framework was established, which included 2 first-level indicators, 10 second-level indicators and 37 third-level indicators, and could reasonably reflect the core contents and performances of TB control at primary TB control institutions.

Analytic hierarchy process (AHP) was a qualitative and quantitative, systematic and hierarchical analysis method, which was widely used in sociological, economics, management, engineering and other fields [19–22]. The indicators framework for comprehensively evaluating the performances in primary TB control institutions being established in this study was a multi-level and multi-indicators composite system, and the importance of each level and indicator needs to be determined scientifically. Using AHP method to determine the weights of indicator was to comprehensively calculate the weight coefficient of indicator by comparing the relative importance of each indicator at the same level, and the reliability and validity of the weights of indicators being determined were ensured by checking the consistency of the judgment matrix. In this study, the result of AHP was based on the score of the importance of indicators and the group decision of experts, which had good rationality and differentiation.

The indicators framework for comprehensively evaluating the performances in primary TB control institutions in this study took into account both objective performances and subjective effects, and covered all aspects of the work, which fully reflected the characteristics and focused of the new TB control model of trinity form.

From the view of the contents covered by the indicators, the objective performances included: discovery, report, registration, treatment and management of patients, and health education, quality control, training and coordination, a total of 8 aspects, that fully reflected the work flow of the new TB control model of trinity form. Among them, discovery and treatment of patients contain more third-level indicators, there were 7 third-level indicators and 6 third-level indicators, respectively, those were the focus works of the primary TB control institutions. It was in line with the basic TB control strategy in China, that was to discovery and treatment of patients timely, to control the source of infection, and to reduce the spread of TB in the population [23]. These two aspects were also the focus of study on TB control performance, which were used to evaluate the achievement of the objectives of national TB control plan [24–27]. Quality control was very important for TB control, which contained 9 third-level indicators, the content involved laboratory internal quality control, diagnosis and treatment process, infection control and adverse drug reactions reporting, and it could comprehensively evaluate the overall quality and ability of TB control. The subjective effects included: the satisfaction rate of medical staff and patients, each aspect containing a third-level indicator. Those two aspects were used to evaluate the satisfaction about the treatment of work for medical staff and the costs of treatment for patients. It was in line with the current situation of the two major challenges that insufficient human resources and heavy disease economic burden of patients, which were the mainly limitations to the performance of the primary TB control institution in Guangxi and the other regions of China [28].

From the view of the feasibility of indicators, each indicator had a clear definition and data sources, the data sources of objective performances came from National TB Management Information System and the daily work record for TB control; the data of subjective effect could be obtained by questionnaire survey in practical work.

From the view of the weights of indicators, among the first-level indicators, the objective performances had a high weight of 0.5444, which indicating that evaluating the specific tasks achievement was the main point of the performance evaluation. Among the second-level indicators, in the aspect of objective performances, indicators with the weights ranked in the top three were discovery, treatment and management of patients, that the combined weights were 0.0743, 0.0732, 0.0679 in turn; In the aspect of subjective effects, the weight of the satisfaction of the patients was higher of 0.2284, which indicated the concept of the patient-centered in the work of primary TB control institutions [29]. Among the third-level indicators, indicators with the highest weight in each aspect were as follows respectively: examination rate of sputum smear, timely reporting rate, registration rate of therapy, receiving rate of treatment, rate of regular medication, completion rate of health education, qualified rate of sputum smear, completion rate of training tasks; satisfaction rate of medical staff about the treatment of work; and satisfaction rate of patients about the costs of treatment, those indicators indicated the top priority of work, and had a great significance for effectively evaluating the performance of TB control in primary TB control institutions.

### Prospects for the next work

The reliability and operability of the indicators framework for comprehensively evaluating the performances in primary TB control institutions in this study needed to be tested through empirical research. Therefore, the next work was to apply the indicators framework to the TB work practice in Guangxi, test the reliability and validity of the indicators framework, and continuously improve based on the needs and development of practice, so as to ensure the objectivity, authenticity and accuracy of the indicators.

## Conclusion

In summary, this study focused on the performance of primary TB control institutions, following the scientific, comprehensive and practical principles, based on a large number of literature and policy guidelines at home and abroad, and an indicators framework fit for primary TB control institutions was established, which can provide a scientific and reasonable evaluation tool for comprehensively evaluating the performance of primary TB control institutions under the new TB control model of trinity form in Guangxi. In addition, our study conducted consultation with experts in Guangxi, so the results maybe more suitable for the work practice in Guangxi, and could also provide reference for the other regions in China.

## Supplementary Information

**Additional file 1: Table S1.** The initial indicators framework.

## Data Availability

The datasets generated and/or analyzed in the current study are not publicly available, due to the personal privacy of the consulting experts, such as their names, identification numbers, work addresses and so on, but are available from the corresponding author on reasonable request.
