# Supplementary material for: How to establishing an indicators framework for evaluating the performances in primary TB control institutions under the new TB control model? Based on a Delphi study conducted in Guangxi, China
Source: BMC Public Health. 2022 Dec 27;22:2431. doi: 10.1186/s12889-022-14865-4 (PMC9792919; doi:10.1186/s12889-022-14865-4)
Supplement: Supplementary file 1 — Additional file 1: Table S1. The initial indicators framework. [file 12889_2022_14865_MOESM1_ESM.docx]

The initial indicators framework: including 2 first-level indicators,10 second-level indicators and 69 third-level indicators. Please see table 4.

| Table S1 The initial indicators framework | | |
| --- | --- | --- |
| First level indicators  （2 indicators） | Second level indicators  （10 indicators） | Third level indicators  （69 indicators） |
| Objective performances | Discovery of patients | Examination rate of sputum smear |
|  |  | Examination rate of three sputum smear |
|  |  | Detection rate of positive patients |
|  |  | Screening rate of drug resistance |
|  |  | Screening rate of close contacts |
|  |  | Referral rate of suspected patients over the hospital |
|  |  | Referral rate of suspected patients within the region |
|  |  | Overall attendance rate of suspected patients |
|  |  | Follow-up rate for patients or suspected patients |
|  |  | Screening rate of close contacts |
|  |  | Screening rate of the older |
|  |  | Screening rate of people with diabetes |
|  | Report of patients | Timely reporting rate |
|  |  | Missing reporting rate |
|  | Registration of patients | Registration rate of first visit |
|  |  | Standard rate of registration |
|  |  | Registration rate of tracking |
|  |  | Registration rate of referral |
|  |  | Registration rate of therapy |
|  |  | Registration rate of outpatients |
|  |  | Registration rate of screening |
|  |  | Complete rate of registration |
|  |  | Registration rate of positive patients |
|  |  | Timely rate of registration |
|  | Treatment of patients | Standard diagnosis rate of negative patients |
|  |  | Diagnosis expert team for negative patients |
|  |  | Utilization rate of the standard treatment scheme |
|  |  | Receiving rate of treatment |
|  |  | Cure rate of positive patients |
|  |  | Successful treatment rate of patients |
|  |  | Treatment interruption rate of patients |
|  |  | Treatment failure rate of patients |
|  |  | Mortality rate of patients |
|  | Management of patients | Rate of regular medication |
|  |  | Rate of scheduled return visits |
|  |  | Completion rate of the first follow up |
|  |  | Completion rate of follow up |
|  |  | Attendance rate of tracking |
|  | Health Education | Plan making rate of health education |
|  |  | Completion rate of health education |
|  |  | Registration rate of health education |
|  | Quality Control | Completion rate of technical guidance |
|  |  | Establish of laboratory internal quality control |
|  |  | Set up full-time laboratory personnel |
|  |  | Qualified rate of sputum smear |
|  |  | Standard rate of the experimental records |
|  |  | Laboratory complies with biosafety |
|  |  | Outpatient procedures is formulate |
|  |  | Establishment of infection control system |
|  |  | Inpatient ward is divided |
|  |  | Set up full-time infection control personnel |
|  |  | Funds for infection control is allocated |
|  |  | Standard rate of drugs registration |
|  |  | Set up a separate medicine cabinet |
|  |  | Drug storage is up to standard |
|  |  | Standard rate of drug distribution |
|  |  | Compliance rate of drug account and substance |
|  |  | Adverse drug reactions reporting is established |
|  | Training and Coordination | Completion rate of training tasks |
|  |  | Completion rate of supervising tasks |
|  |  | Completion rate of work meeting tasks |
|  |  | Coordination mechanism is established |
|  |  | Annual training rate for Rural doctors |
|  |  | Annual training rate for physicians |
| Subjective effect | Satisfaction rate of medical staff | Satisfaction rate about the environment of work |
|  |  | Satisfaction rate about the treatment of work |
|  | Satisfaction rate of patients | Satisfaction rate about the treatment |
|  |  | Satisfaction rate about health care workers |
|  |  | Satisfaction rate about the costs of treatment |

|  |  |
| --- | --- |
|  |  |
|  |  |
|  |  |
|  |  |
|  |  |
|  |  |
|  |  |
|  |  |
|  |  |
|  |  |
|  |  |
|  |  |
|  |  |
|  |  |
|  |  |
|  |  |
|  |  |
|  |  |
|  |  |
|  |  |
|  |  |
|  |  |
|  |  |
|  |  |
|  |  |
|  |  |
|  |  |
|  |  |
|  |  |
|  |  |
|  |  |
|  |  |
|  |  |
|  |  |
|  |  |
|  |  |
|  |  |
|  |  |
|  |  |
|  |  |
|  |  |
|  |  |
|  |  |
|  |  |
|  |  |
|  |  |
